# Supplementary material for: Dynalign II: common secondary structure prediction for RNA homologs with domain insertions
Source: Nucleic Acids Res. 2014 Nov 21;42(22):13939–48. doi: 10.1093/nar/gku1172 (PMC4267632; doi:10.1093/nar/gku1172)
Supplement: SUPPLEMENTARY DATA [file supp_gku1172_nar-02021-z-2014-File012.zip › manual/javadoc/RNAstructure_java_drawing/source/dialogs/package-summary.html]

RNAstructure\_java\_drawing.source.dialogs


JavaScript is disabled on your browser.


- Overview
- Package
- Class
- Tree
- Index
- Help

- Prev Package
- Next Package

- Frames
- No Frames

- All Classes

# Package RNAstructure\_java\_drawing.source.dialogs

A package that holds all Java drawing dialogs.

See: Description

- Class Summary

  | Class | Description |
  | --- | --- |
  |  |  |
  | --- | --- |
  | ImageDialog | A printable and zoomable dialog that holds drawn images. |
  | PlotDialog | A dialog that holds dot plot images. |
  | StructureDialog | A dialog that holds structure images. |


## Package RNAstructure\_java\_drawing.source.dialogs Description

A package that holds all Java drawing dialogs.
  
  
The drawing dialogs are in a hierarchy, with the base class handling all common dialog tasks, and the subclasses handling specific painting routines.
Subclasses also contain other methods besides painting routines that contain specialized GUI actions.

- Overview
- Package
- Class
- Tree
- Index
- Help

- Prev Package
- Next Package

- Frames
- No Frames

- All Classes
